# A Novel *MTTK* Gene Variant m.8315A>C as a Cause of MERRF Syndrome

**DOI:** 10.3390/genes13071245

**Published:** 2022-07-14

**Authors:** Hana Štufková, Hana Kolářová, Kateřina Lokvencová, Tomáš Honzík, Jiří Zeman, Hana Hansíková, Markéta Tesařová

**Affiliations:** Department of Pediatrics and Inherited Metabolic Disorders, First Faculty of Medicine, Charles University, and General University Hospital in Prague, 128 08 Prague, Czech Republic; hana.stufkova@lf1.cuni.cz (H.Š.); hana.kolarova@vfn.cz (H.K.); katerina.lokvencova@vfn.cz (K.L.); tomas.honzik@vfn.cz (T.H.); jzem@lf1.cuni.cz (J.Z.); hana.hansikova@lf1.cuni.cz (H.H.)

**Keywords:** mtDNA, *MTTK* gene, OXPHOS, heteroplasmy, m.8315A>C

## Abstract

In this study, we report on a novel heteroplasmic pathogenic variant in mitochondrial DNA (mtDNA). The studied patient had myoclonus, epilepsy, muscle weakness, and hearing impairment and harbored a heteroplasmic m.8315A>C variant in the *MTTK* gene with a mutation load ranging from 71% to >96% in tested tissues. In muscle mitochondria, markedly decreased activities of respiratory chain complex I + III and complex IV were observed together with mildly reduced amounts of complex I and complex V (with the detection of V*- and free F1-subcomplexes) and a diminished level of complex IV holoenzyme. This pattern was previously seen in other *MTTK* pathogenic variants. The novel variant was not present in internal and publicly available control databases. Our report further expands the spectrum of *MTTK* variants associated with mitochondrial encephalopathies in adults.

## 1. Introduction

The analysis of mitochondrial DNA (mtDNA) is an essential step in the diagnosis of patients with mitochondrial disease (MD), and although variations in nuclear genes are increasingly being reported, mtDNA sequencing should precede more complex genomic analysis, especially in adults with suspected MD. Since their first description in 1990, the number of pathogenic variants in mitochondrial tRNA genes has steadily increased [1,2], and associations with a striking variety of clinical features have been reported [3]. Mitochondrial disease can present in infancy or adulthood in a multisystemic or highly tissue-specific manner. Many organ systems can be affected in varying combinations based on the heteroplasmy levels and energy demand in particular tissues. The *MTTK* gene, coding for mitochondrial tRNA-Lys, is the second most common site of mutations in mtDNA, and 28 pathogenic variants have been described [4]. Mitochondrial tRNA-Lys, similarly to other 21 tRNAs encoded by mtDNA, is essential for mitochondrial translation; thus, its mutations lead to a combined deficiency of oxidative phosphorylation (OXPHOS) complexes. Classically, patients present ataxia, myoclonus, or epilepsy, indicating CNS involvement and myopathy [5,6].

## 2. Materials and Methods

### 2.1. Patient

The patient, now a 39-year-old woman, is the only child of healthy, unrelated parents, and her early development was uneventful. Since the age of 10 years, the patient reported having increased fatigue and slightly altered postural stability. However, until her early adulthood, the patient’s state was rather unremarkable, and she was able to complete her master’s degree. At age of 30, the patient developed focal sensory seizures with numerous multi-colored photopsias arising from the right occipital lobe and lasting several hours. She felt the progression of both fatigue and exercise intolerance and further balance deterioration. At the age of 35, focal motoric seizures in the left hemisphere occurred. She was never well-stabilized and required repeated hospitalizations due to repeated migrating myoclonias. During an intercurrent Candida sepsis, the seizures progressively increased in frequency and evolved to generalized convulsive status epilepticus refractory to acute first- and second-line antiepileptic drugs. Currently, she suffers from epilepsy partialis continua with daily myoclonic seizures in her left abdominal wall. At the latest clinical evaluation, she was severely atactic and dysarthric, and she reported occasional difficulty swallowing. There was moderate proximal and distal weakness in all extremities and postictal hypoesthesia in the left side of the face, left arm, and right leg. A recent audiology examination revealed mild mixed bilateral hearing impairment. The blood test results were normal, including serum CK and lactic acid concentrations, but her serum alanine level was mildly increased (538 μmol/L; controls < 500). Mild hyperlactaciduria was observed (76 mmol/mol of creatinine; controls < 30).

### 2.2. Mitochondrial DNA Sequencing

Total genomic DNA was isolated from blood, muscle biopsy, buccal swab cells, and hair follicles using the following kits: the Gentra Puregene Blood Kit, QIAamp DNA Mini Kit, and QIAamp DNA Micro Kit, respectively (all Qiagen, Hilden, Germany). For mutation analysis, the SeqCap EZ Design: Mitochondrial Genome Design (Roche NimbleGen, Pleasonton, CA, USA) enrichment kit was used for the preparation of the sequencing library, followed by analysis using the MiSeq (Illumina, San Diego, CA, USA) system, specifically, the MiSeq Reagent Sequencing kit v3. The revised Cambridge Reference Sequence (NC_012920) was used for variant annotation.

### 2.3. Mitochondria Isolation

A sample obtained by muscle biopsy was transported on ice (at 4 °C), and mitochondria were isolated immediately according to standard differential centrifugation procedures [7] in a buffer containing 150 mM KCl, 50 mM Tris/HCl, 2 mM EDTA, and 2 μg/mL aprotinin (pH 7.5) at 4 °C [8].

### 2.4. MEGS Analysis

The analysis of the mitochondrial energy-generating system (MEGS) capacity in fresh postnuclear muscle supernatant was determined by measuring the oxidation rates of 14C-labeled pyruvate malate and succinate, donors and acceptors of Acetyl-CoA, and inhibitors of TCA cycle according to Janssen et al. [9] using ten different incubations.

### 2.5. Spectrophotometry

The activities of respiratory chain complexes (complex I—NADH:coenzyme Q oxidoreductase, CI, EC 1.6.5.3; complex I + III—NADH:cytochrome c oxidoreductase, CI + III; complex II—succinate-coenzyme Q oxidoreductase, CII, EC 1.3.5.1; complex II + III—succinate:cytochrome c oxidoreductase, CII + III; complex III—coenzyme Q:cytochrome c oxidoreductase, CIII, EC 7.1.1.8; complex IV—cytochrome c oxidase, CIV, EC 1.9.3.1) were measured according to [10]. The activity of citrate synthase (CS, EC 2.3.3.1), serving as the control enzyme to avoid assay variability, was measured according to [11]. Protein concentrations were measured using the Lowry method.

### 2.6. Electrophoresis

Blue Native Polyacrylamide Gel Electrophoresis (BN-PAGE) separation [12] of mitochondrial membrane complexes on polyacrylamide 6–15% (*w*/*v*) gradient gels (MiniProtean^®^ 3 System; Bio-Rad, Hercules, CA, USA) followed by immunoblot analysis was used to analyze the steady-state levels of the oxidative phosphorylation system complexes. The primary detection of BN-PAGE blots was performed using mouse monoclonal antibodies against the CI subunit NDUFA9 (1:2000), complex II subunit SDH70 protein (1: 20,000), complex III subunit Core 2 (1:20,000), complex IV subunit COX2 (1:10,000), and ATP synthase subunit alpha (1:4000) (Abcam, Cambridge, UK). The immunoblots were detected with peroxidase-conjugated secondary antibodies and SuperSignal West Femto Maximum Sensitivity Substrate (Thermo Fisher Scientific, Waltham, MA, USA) using G:Box (Syngene, Cambridge, UK).

## 3. Results

**Figure 1:**
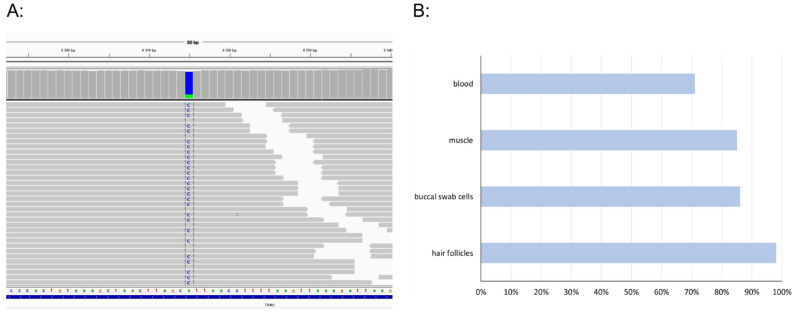
A *MTTK* gene variant m.8315A>C. IGV snapshot of mtDNA sequencing in patient’s muscle (**A**); overview of heteroplasmy levels in patient tissues (**B**).

MtDNA sequencing in blood revealed a heteroplasmic variant m.8315A>C in the *MTTK* gene. To further assess the variant, several tissues, including muscle biopsy, were collected, and mutation loads were determined. The highest level of heteroplasmy was found in hair follicles > 96%, followed by 86% in buccal swab cells, 85% in muscle, and 71% in blood leucocytes (Figure 1).

In a muscle sample obtained from a biopsy of the quadriceps, the analysis of the MEGS capacity indicated disturbed OXPHOS activity with decreased ADP stimulation. The respiratory chain enzyme activities in isolated mitochondria showed normal activities of respiratory chain complexes I and III. Markedly decreased were activities of complexes I + III and IV, as well as complex IV to the citrate synthase (CS) ratio in comparison to age-related controls (Table 1). Interestingly, the activities of respiratory chain complexes II and II + III were slightly increased, suggesting a compensation effect. However, no structural changes or RRF- or COX-negative fibers were detected.

**Figure 2:**
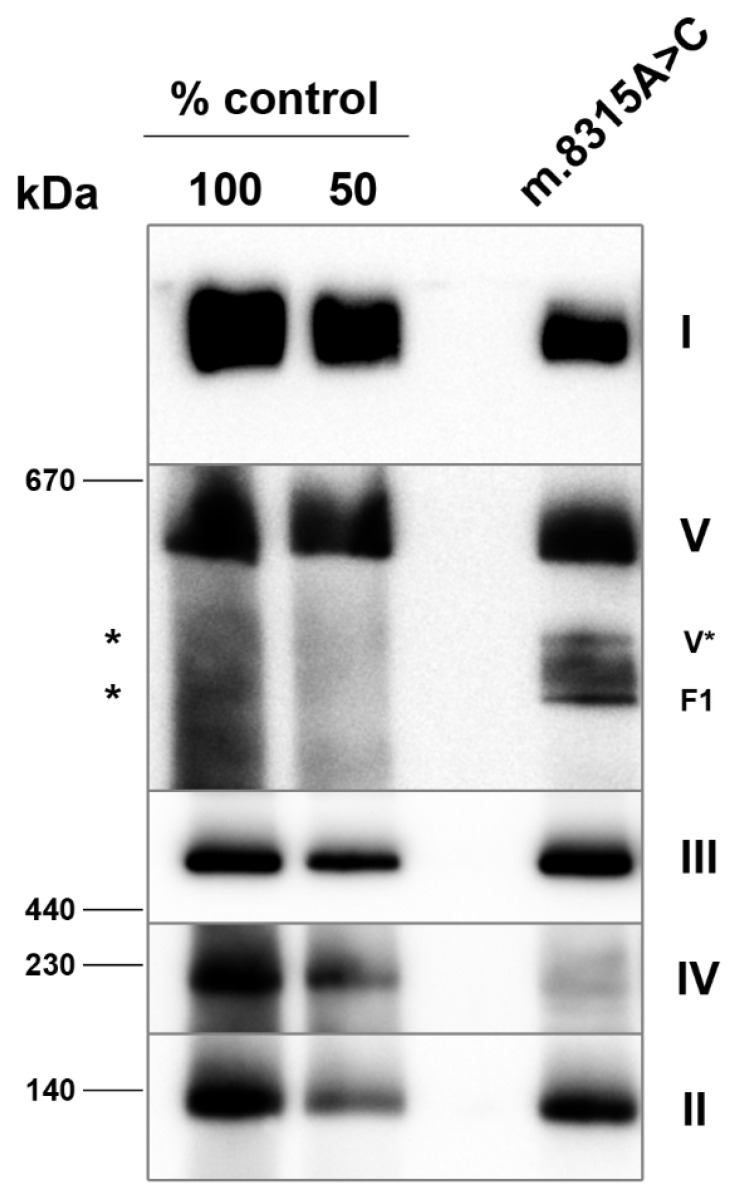
Analysis of the assembly of OXPHOS complexes by immunoblotting of BN-PAGE in muscle mitochondria. BN-PAGE of lauryl maltoside-solubilized mitochondria was electroblotted onto PVDF membranes and probed with monoclonal antibodies that detect the native forms of the OXPHOS complexes. Two aliquots of control mitochondria corresponding to the indicated dilutions of control samples were loaded on the same gels. I—complex I; II—complex II; III—complex III; IV—complex IV; V—complex V; V*—complex V subcomplex composed of F1-ATPase with several c-subunits; F1—F1-ATPase; *-expected migration of complex V subcomplexes.

The separation of muscle mitochondrial proteins using BN-PAGE followed by Western blotting and immunodetection revealed mildly reduced amounts of complex I (40% of the control) and complex V (approx. 50% of the control) with accumulated sub-complexes, most likely V* (F1-ATPase with several c-subunits) and F1-ATPase (α and β subunits hexamer with central stalk subunits). The steady-state level of complex IV holoenzyme was diminished in patient mitochondria (approx. 20% of the control, Figure 2).

## 4. Discussion

Mitochondrial tRNA pathogenic variants are associated with a wide range of clinical phenotypes. Over the years, almost 30 disease variants in the *MTTK* gene have been identified and their pathogenicity more or less assessed. We found an unreported heteroplasmic variant m.8315A>C localized between the D-stem and AC-stem of mitochondrial tRNA-Lys. Using ACMG/AMP criteria adapted for mitochondrial variants [13], the m.8315A>C variant was classified as a variant of unknown significance since it is absent from controls (PM2), the phenotype suggests a single gene etiology (PP4), and it was classified as likely benign by in silico analyses (BP4). Nonetheless, we provide several key arguments supporting the pathogenicity of the m.8315A>C variant. First, despite the linker between D- and AC- stems not having been previously predicted as a mutation hotspot among mt-tRNAs [14], based on *MTTK* sequence alignment, there is only a single purine nucleotide, either A or G, found in this position among 40 species [15]. Thus, the substitution of adenine for cytosine at position m.8315 may disturb interactions stabilizing the secondary structure of mt-tRNA-Lys. The purine nucleotides are conserved in the linker between the D-stem and AC-stem of other five mt-tRNAs (mt-tRNA-Glu, mt-tRNA-Ile, mt-tRNA-Cys, mt-tRNA-Phe, and mt-tRNA-Tyr) [15]. Second, the m.8315A>C variant was absent from the available databases, Mitomap [4], GnomAD [16], and Helix [17], as well as our internal one. Interestingly, m.8315A>G occurred with a frequency of 0.002–0.037% in public databases, which is in accordance with the conservation of the purine nucleotide in this position. Third, the heteroplasmy levels differed in the tested tissues of the patient, with the lowest mutation load observed in the blood. Unfortunately, no maternal relatives were available for mutation testing since the patient has no siblings or children, and her mother is deceased. Fourth, the muscle biopsy provided significant support for the pathogenicity of the m.8315A>C variant. Despite the absence of ragged-red fibers, which is not unique for MERRF (myoclonic epilepsy with ragged-red fibres) patients [18], combined OXPHOS complexes deficiency was apparent in the patient´s muscle mitochondria. Based on the activity measurement and analysis of the steady-state levels of OXPHOS complexes, complex IV was the most severely affected, compared to a milder complex I and complex V deficiency. We observed this pattern in our previous study analyzing the impact of two *MTTK* genes’ (m.8363G>A and m.8344A>G) and one *MTTL1* gene’s (m.3243A>G) pathogenic variants on OXPHOS complexes in muscle, heart, liver, and brain mitochondria [19]. In muscle, both *MTTK* gene variants resulted in profoundly decreased levels of complex IV, with similar or milder decreases in complex I levels with markedly reduced levels of the complex V holoenzyme with accumulated sub-complexes containing F1-ATPase. Interestingly, each of the *MTTK* variants, including the novel m.8315A>C variant, are localized in different secondary structures of mitochondrial tRNA-Lys (m.8315—linker of D-stem and AC-stem, m.8344—T-loop, and m.8363—acceptor stem), which results in the same pattern of combined OXPHOS complex deficiency (diminished CIV levels, with a profound decrease in CI and CV, and accumulated CV sub-complexes), which is distinct from the *MTTL1* gene variant [19].

## 5. Conclusions

Our report provides solid evidence supporting the pathogenicity of a novel heteroplasmic variant, m.8315A>C, and further expands the spectrum of *MTTK* gene variants associated with mitochondrial encephalopathies in adults. It also highlights the substantial role of muscle biopsies in the evaluation of the pathogenicity of novel mtDNA variants. However, additional reports are necessary to definitively confirm the pathogenicity of the m.8315A>C variant.

## Figures and Tables

**Table 1:** Activities of respiratory chain complexes in muscle mitochondria.

	Patient	Controls (n = 26)
	(nmol/min/mg Protein)	
Complex I	262.1	118–282
Complex I + III	**50.7**	100–287
Complex II	124.8	28–94
Complex II + III	320	97–267
Complex III	620.8	321–640
Complex IV	**112.9**	825–1500
Citrate Synthase (CS)	573.3	528–938
Complex IV/CS	**0.20**	1.16–2.13

## Data Availability

Not applicable.

## References

[1] Shoffner J.M., Lott M.T., Lezza A.M., Seibel P., Ballinger S.W., Wallace D.C. (1990). Myoclonic epilepsy and ragged-red fiber disease (MERRF) is associated with a mitochondrial DNA tRNA(Lys) mutation. Cell.

[2] Richter U., McFarland R., Taylor R.W., Pickett S.J. (2021). The molecular pathology of pathogenic mitochondrial tRNA variants. FEBS Lett..

[3] DiMauro S., Schon E.A., Carelli V., Hirano M. (2013). The clinical maze of mitochondrial neurology. Nat. Rev. Neurol..

[4] MITOMAP: A Human Mitochondrial Genome Database 2019. http://www.mitomap.org.

[5] Blakely E.L., Alston C.L., Lecky B., Chakrabarti B., Falkous G., Turnbull D.M., Taylor R.W., Gorman G.S. (2014). Distal weakness with respiratory insufficiency caused by the m.8344A>G “MERRF” mutation. Neuromuscul. Disord..

[6] Mancuso M., Orsucci D., Angelini C., Bertini E., Carelli V., Comi G.P., Minetti C., Moggio M., Mongini T., Servidei S. (2013). Phenotypic heterogeneity of the 8344A>G mtDNA “MERRF” mutation. Neurology.

[7] Makinen M.W., Lee C.P., Shy G.M. (1968). Microanalysis of cytochrome content, oxidative and phosphorylative activities of human skeletal muscle mitochondria. Neurology.

[8] Jesina P., Tesarova M., Fornuskova D., Vojtiskova A., Pecina P., Kaplanova V., Hansikova H., Zeman J., Houstek J. (2004). Diminished synthesis of subunit a (ATP6) and altered function of ATP synthase and cytochrome c oxidase due to the mtDNA 2 bp microdeletion of TA at positions 9205 and 9206. Biochem. J..

[9] Janssen A.J., Trijbels F.J., Sengers R.C., Wintjes L.T., Ruitenbeek W., Smeitink J.A., Morava E., van Engelen B.G., van den Heuvel L.P., Rodenburg R.J. (2006). Measurement of the energy-generating capacity of human muscle mitochondria: Diagnostic procedure and application to human pathology. Clin. Chem..

[10] Rustin P., Chretien D., Bourgeron T., Gerard B., Rotig A., Saudubray J.M., Munnich A. (1994). Biochemical and molecular investigations in respiratory chain deficiencies. Clin. Chim. Acta.

[11] Srere P.A., John M.L. (1969). Citrate synthase: [EC 4.1.3.7. Citrate oxaloacetate-lyase (CoA-acetylating)]. Methods in Enzymology.

[12] Schagger H., Cramer W.A., von Jagow G. (1994). Analysis of molecular masses and oligomeric states of protein complexes by blue native electrophoresis and isolation of membrane protein complexes by two-dimensional native electrophoresis. Anal. Biochem..

[13] McCormick E.M., Lott M.T., Dulik M.C., Shen L., Attimonelli M., Vitale O., Karaa A., Bai R., Pineda-Alvarez D.E., Singh L.N. (2020). Specifications of the ACMG/AMP standards and guidelines for mitochondrial DNA variant interpretation. Hum. Mutat..

[14] Blakely E.L., Yarham J.W., Alston C.L., Craig K., Poulton J., Brierley C., Park S.M., Dean A., Xuereb J.H., Anderson K.N. (2013). Pathogenic mitochondrial tRNA point mutations: Nine novel mutations affirm their importance as a cause of mitochondrial disease. Hum. Mutat..

[15] Putz J., Dupuis B., Sissler M., Florentz C. (2007). Mamit-tRNA, a database of mammalian mitochondrial tRNA primary and secondary structures. RNA.

[16] Laricchia K.M., Lake N.J., Watts N.A., Shand M., Haessly A., Gauthier L., Benjamin D., Banks E., Soto J., Garimella K. (2022). Mitochondrial DNA variation across 56,434 individuals in gnomAD. Genome Res..

[17] Bolze A., Mendez F., White S., Tanudjaja F., Isaksson M., Jiang R., Rossi A.D., Cirulli E., Rashkin M., Metcalf W. (2019). A catalog of homoplasmic and heteroplasmic mitochondrial DNA variants in humans. bioRxiv.

[18] Mancuso M., Petrozzi L., Filosto M., Nesti C., Rocchi A., Choub A., Pistolesi S., Massetani R., Fontanini G., Siciliano G. (2007). MERRF syndrome without ragged-red fibers: The need for molecular diagnosis. Biochem. Biophys. Res. Commun..

[19] Fornuskova D., Brantova O., Tesarova M., Stiburek L., Honzik T., Wenchich L., Tietzeova E., Hansikova H., Zeman J. (2008). The impact of mitochondrial tRNA mutations on the amount of ATP synthase differs in the brain compared to other tissues. Biochim. Biophys. Acta.

